# Impacts of inbreeding on bumblebee colony fitness under field conditions

**DOI:** 10.1186/1471-2148-9-152

**Published:** 2009-07-02

**Authors:** Penelope R Whitehorn, Matthew C Tinsley, Mark JF Brown, Ben Darvill, Dave Goulson

**Affiliations:** 1 School of Biological and Environmental Sciences, University of Stirling, Stirling, UK; 2 Department of Zoology, School of Natural Sciences, Trinity College Dublin, Dublin 2, Ireland; 3 School of Biological Sciences, Royal Holloway, University of London, Egham, Surrey, UK

## Abstract

**Background:**

Inbreeding and the loss of genetic diversity are known to be significant threats to small, isolated populations. Hymenoptera represent a special case regarding the impact of inbreeding. Haplodiploidy may permit purging of deleterious recessive alleles in haploid males, meaning inbreeding depression is reduced relative to diploid species. In contrast, the impact of inbreeding may be exacerbated in Hymenopteran species that have a single-locus complementary sex determination system, due to the production of sterile or inviable diploid males. We investigated the costs of brother-sister mating in the bumblebee *Bombus terrestris*. We compared inbred colonies that produced diploid males and inbred colonies that did not produce diploid males with outbred colonies. Mating, hibernation and colony founding took place in the laboratory. Once colonies had produced 15 offspring they were placed in the field and left to forage under natural conditions.

**Results:**

The diploid male colonies had a significantly reduced fitness compared to regular inbred and outbred colonies; they had slower growth rates in the laboratory, survived for a shorter time period under field conditions and produced significantly fewer offspring overall. No differences in success were found between non-diploid male inbred colonies and outbred colonies.

**Conclusion:**

Our data illustrate that inbreeding exacts a considerable cost in *Bombus terrestris *through the production of diploid males. We suggest that diploid males may act as indicators of the genetic health of populations, and that their detection could be used as an informative tool in hymenopteran conservation. We conclude that whilst haplodiploids may suffer less inbreeding depression than diploid species, they are still highly vulnerable to population fragmentation and reduced genetic diversity due to the extreme costs imposed by the production of diploid males.

## Background

The genetic health of populations is increasingly viewed as one of the most important factors in maintaining fitness in an uncertain and changing environment [1]. It is well established that inbreeding depression in diploid organisms significantly increases the risk of extinction [2]. By contrast, haplodiploid organisms have often been assumed to suffer less inbreeding depression as deleterious recessive mutations were thought to be purged through the haploid males [3]. However, some authors have challenged this assumption, partly because purging may not be effective against female sex-limited traits, such as hibernation survival and fecundity [4].

Haplodiploids may suffer further genetic costs of inbreeding due to their single-locus complementary sex determination (sl-CSD) system, which is ancestral to the haplodiploid Hymenoptera. Under this system, individuals heterozygous at the polyallelic sex-determining locus develop into diploid females and hemizygotes develop into haploid males. When a diploid individual is homozygous at the sex locus a diploid male is produced. The frequency of diploid males depends on the number of CSD alleles and so they are rarely produced in large outbreeding populations because many alleles are maintained by negative frequency-dependent selection [5,6]. However, genetic drift in small populations is expected to increase diploid male production (DMP) by reducing CSD allelic richness [7].

Diploid males represent significant fitness costs, primarily through their inviability or sterility [8,9]. In a few species, diploid males can produce diploid sperm and mate, but this invariably results in sterile triploid progeny so the costs are merely deferred by a generation [10]. In social insects further costs of diploid males are apparent, as they replace 50% of the female workers and do not contribute to colony productivity [5]. This has been shown to slow the rate of colony growth in *Bombus atratus*, under laboratory conditions [11] and result in high mortality of founding queens in the fire ant *Solenopsis invicta *[12]. Recent modelling has demonstrated that DMP can initiate a rapid extinction vortex and suggests that haplodiploids are more prone to extinction than previously supposed [13].

The study of genetic diversity and inbreeding in bumblebees is currently of particular importance as many species have been suffering from significant population declines and range contractions [14,15]. This has been attributed predominantly to the intensification of agriculture and the associated loss of flower rich meadows and other habitats on which bumblebees depend [16-19]. The remaining populations of rare species have become fragmented, genetically isolated and suffer from a loss of genetic diversity. They are now susceptible to inbreeding depression, with serious implications for their persistence [20-22].

The genetic consequences of population fragmentation and isolation are exacerbated in bumblebees as a number of factors predispose them to low levels of heterozygosity and hence inbreeding. Firstly, as haplodiploids, there are only 75% as many gene copies in any one generation compared to diplodiploid organisms, hence reducing the effective population size [23]. Secondly, the effective population size of bumblebees is reduced still further by their social nature, as it is determined by the number of successful nests in an area and not by the number of more abundant sterile workers [24]. Finally, the majority of bumblebee species are monandrous [25,26]. This increases their susceptibility to inbreeding compared to polyandrous species, which effectively have more breeding individuals per generation [6] and which in some instances can avoid the costs of negative genetic incompatibility through postcopulatory selective fertilization [27]. Whilst small effective population size in haplodiploids may not result in inbreeding depression *per se *it will decrease CSD allelic richness, which in turn will increase diploid male production.

Investigations into the effects of inbreeding in bumblebees have had varying outcomes. Under laboratory conditions one generation of brother-sister mating in *Bombus terrestris *had no effect on immune defence or body size [28,29]. However, a similar experiment found that inbreeding did have a significant negative effect on colony size, whereas the impact of inbreeding on other fitness traits was highly variable across maternal genotypes [29]. Additionally, when *B. terrestris *queens were sib-mated for several generations, a negative effect on queen fecundity and colony size was observed [30]. The cost of diploid male production is unclear: Duchateau *et al. *[5] found that the growth rate of laboratory diploid male colonies of *B. terrestris *was not significantly affected, yet Plowright & Pallett [11] found diploid male colonies of *Bombus atratus *had a significantly slower growth rate, albeit with a very small sample size. Diploid males have been observed in rare and threatened bumblebee species in the wild [20,22], so the true costs of their production are important to ascertain.

This study aimed to determine the costs of brother-sister mating in the bumblebee *Bombus terrestris*, specifically focusing on survival and growth in field conditions and the fitness of diploid male colonies. Young *B. terrestris *gynes were mated in the laboratory with either their brothers or with un-related males. Their survival during hibernation was recorded and those queens that established colonies in the laboratory generated three experimental treatments:

1) Sib-mated queens not producing diploid male offspring (Inbred treatment)

2) Sib-mated queens producing diploid male offspring (Diploid male treatment)

3) Outbred queen colonies (Outbred treatment)

The rate of growth of these colonies was measured and once they had produced 15 offspring they were placed in the field. The development and survival of these colonies were followed throughout a summer season to demonstrate the costs of inbreeding and DMP in a natural setting.

## Results

### Hibernation survival

**Figure 1:**
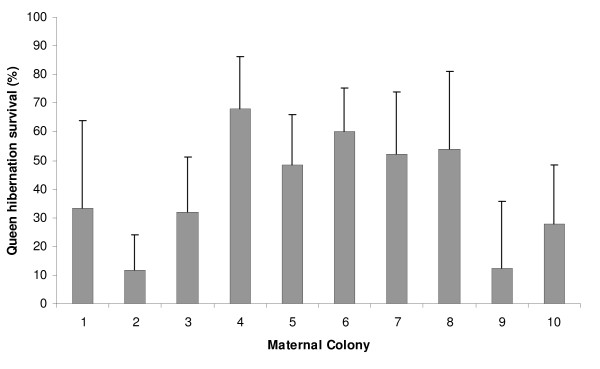
**Hibernation survival for experimental queens from each maternal colony**. The probability of surviving differed significantly between maternal colonies (P < 0.0001). Sample size within each maternal colony ranged from 8 to 40 and error bars show 95% shortest unbiased confidence limits.

In total 93 queens (43.7%) survived the hibernation period and subsequent 72 hours. The probability of surviving hibernation was significantly affected by the maternal family line (χ^2^_9, _= 31.84; P < 0.0001); survival ranged from 11.5% to 68.0% between maternal colonies (see Figure 1). Mating date was also a significant predictor of hibernation survival; queens mated earlier were more likely to survive (χ^2^_1, _= 19.28, p < 0.0001). There was no difference in survival between queens mated to unrelated males and sib-mated queens (46.34%, n = 82 and 41.98%, n = 131 respectively. χ^2^_1, _= 1.67; P = 0.199).

### Colony foundation

Out of the queens that survived hibernation, 47 produced at least one offspring and were considered to have successfully founded a colony. Of these 47 colonies, 20 were outbred, 17 were inbred and 10 were diploid male colonies. There was no difference between colony founding ability between queens mated to unrelated males and sib-mated queens (57.14%, n = 35 and 50.94%, n = 53 respectively. χ^2^_1, _= 0.326, p = 0.568). Additionally, colony founding ability was not predicted by maternal colony (χ^2^_9, _= 14.25, P = 0.114) or hibernation end date (χ^2^_1, _= 0.78, p = 0.378).

### Colony growth

#### The number of colonies reaching 5 and 15 offspring

**Figure 2:**
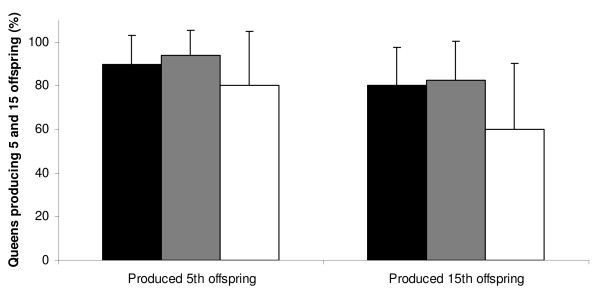
**The percentage of queens producing 5 and 15 offspring according to treatment**. Black bars represent the outbred treatment, grey bars the inbred treatment and white bars the diploid male treatment. No significant difference was found between these values (P = 0.835 for 5 offspring & P = 0.248 for 15 offspring). Sample size within each treatment ranged from 6 to 18 and error bars show 95% shortest unbiased confidence limits.

The probability of a colony growing past the 5 and 15 offspring size thresholds was not influenced by inbreeding status (χ^2^_2, _= 0.36, P = 0.835; χ^2^_2, _= 1.70, P = 0.428 respectively) (Figure 2). Maternal line did not significantly influence the number of colonies reaching 5 and 15 offspring (χ^2^_9, _= 11.42, P = 0.248; χ^2^_9, _= 11.76, P = 0.227 respectively) and neither did hibernation end date (χ^2^_1, _= 0.08, P = 0.779; χ^2^_9, _= 0.54, P = 0.461 respectively).

#### The rate of colony growth

**Figure 3:**
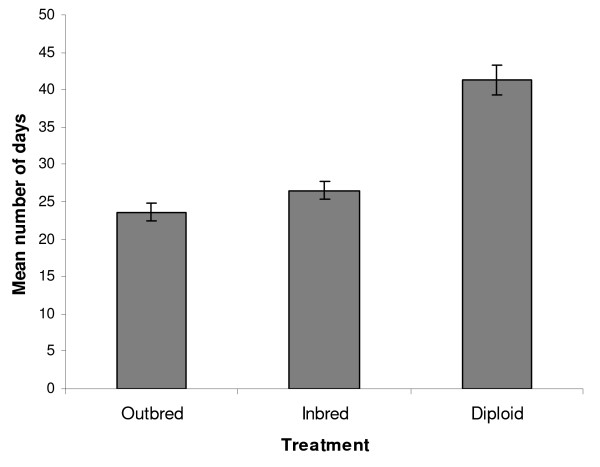
**Mean time from colony foundation to 15^th ^offspring, according to treatment**. Bars show the mean duration of the period between the emergence of the 1^st ^and 15^th ^offspring. Means and their standard errors were predicted from the GLM. This measure of colony growth is significantly slower for the diploid male treatment than either outbred or inbred colonies (P < 0.001, see text).

We assessed the rate of growth of colonies after foundation by recording the time until they crossed three size thresholds: 1, 5 and 15 offspring. The inbreeding treatments did not significantly influence the time taken to reach these sizes, neither did maternal colony origin or hibernation end date (Table 1). However, due to the earlier emergence of offspring in the DMP colonies, the mean interval between emergence of the 1^st ^and 15^th ^offspring was considerably longer for the diploid treatment (41.3 days ± 2.00, n = 6) than for either the inbred (26.51 days ± 1.18, n = 14) or outbred (23.6 days ± 1.21, n = 16) treatments (Figure 3). This variation was highly significant (F_2,25 _= 35.13; P < 0.001). Post hoc tests confirmed that the diploid male treatment differed from both the others (P < 0.0001), but that no difference existed between the inbred and outbred colonies (P > 0.246). Maternal colony also influenced the number of days between the emergence of the 1^st ^and 15^th ^offspring (F_8,25 _= 6.35, p < 0.001).

**Table 1:** Output of GLM for the rate of colony growth

	Days to 1^st ^offspring		Days to 5^th ^offspring		Days to 15^th ^offspring		Days from 1^st ^to 15^th ^offspring	
				
	F	P	F	P	F	P	F	P
								
Inbreeding Treatment	1.70 (2, 46)	0.194	0.75 (2, 39)	0.480	0.62	0.545	35.13 (2, 25)	< 0.001
								
Maternal Colony	1.68 (9, 34)	0.133	1.30 (8, 30)	0.281	1.70 (8, 24)	0.149	6.35 (8, 25)	< 0.001
								
Hibernation end date	2.03 (1, 34)	0.164	1.37 (1, 30)	0.251	3.48 (1, 24)	0.074	2.09 (1, 24)	0.161

The rate of colony growth is represented by the number of days from the hibernation end date to the emergence of the 1^st^, 5^th ^and 15^th ^offspring and the number of days to the emergence of the 15^th ^offspring from the 1^st ^offspring, respectively, with respect to inbreeding treatment, maternal colony and the co-variate hibernation end date. Degrees of freedom are given in parentheses.

### Survival and growth under field conditions

#### Survival in the field

**Figure 4:**
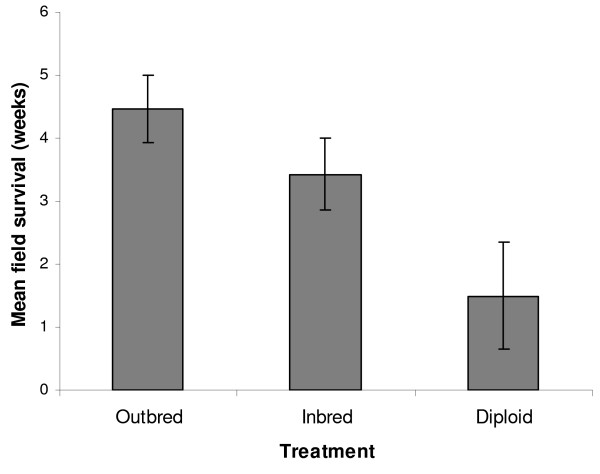
**The mean number of weeks colonies survived under field conditions according to treatment**. Bars represent the least square means and their standard errors as predicted by the GLM. Diploid male colonies survived significantly fewer weeks than the outbred colonies (P < 0.05, see text).

The diploid male colonies survived for a shorter time period under field conditions compared to the outbred and regular inbred colonies; a mean of only 1.5 ( ± 0.86) weeks, compared to means of 4.5 ( ± 0.54) and 3.4 ( ± 0.56) weeks respectively (F_2,32 _= 4.33, p < 0.05) (Figure 4). Post hoc tests revealed the outbred and diploid male treatments were significantly different (p < 0.02); no significant difference existed for other pairwise comparisons (inbred-outbred P = 0.388, inbred-diploid male P = 0.159). Maternal line and field placement date did not cause significant variation in field survival duration (F_7,24 _= 1.19, p = 0.345 and F_1,31 _= 3.49, p = 0.071 respectively).

#### Colony growth in the field

**Figure 5:**
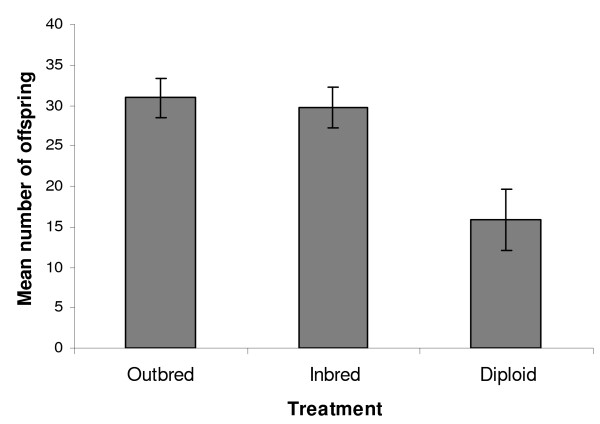
**The mean total number of offspring produced by colonies in the field according to treatment**. Bars represent least square means and their standard errors as predicted by the GLM. Diploid male colonies produced significantly fewer offspring than both the outbred and the regular inbred colonies (P < 0.01, see text).

The number of offspring a colony produces is a major determinant of colony fitness. All colonies had 15 offspring when placed into the field. Outbred and inbred colonies continued to grow under field conditions, producing total means of 30.9 ( ± 2.42) and 29.7 ( ± 2.50) offspring each. However, diploid male colonies produced very few additional offspring in the field, reaching a mean of only 15.8 ( ± 3.82) offspring. This striking variation between inbreeding treatments was significant (F_2,32 _= 6.03, p < 0.01) (Figure 5). Post hoc tests confirmed that diploid male colonies differed significantly from both the outbred and inbred treatments (p = 0.006 & p = 0.013 respectively); the difference in mean size between outbred and inbred colonies was not significant (p = 0.935). Maternal colony and field placement date did not significantly influence final colony size (F_7,24 _= 1.43, p = 0.241; F_1,31 _= 1.39, p = 0.248 respectively).

## Discussion

For the first time we demonstrate that brother-sister mating in *B. terrestris *exacts high costs under field conditions through the production of diploid males. A number of fitness parameters were negatively affected by diploid male production, including colony growth rate, total offspring production and colony survival, but no significant effects of inbreeding in the absence of diploid male production were detected.

The costs of diploid male production were first evident whilst colonies were growing in the laboratory, where the number of days between the emergence of the 1^st ^and 15^th ^offspring was considerably greater for the diploid male colonies. This slower growth rate presumably occurs because colony resources are diverted away from the production of industrious female workers; diploid males are idle within the colony and so the workforce is approximately halved, resulting in less brood care and slower growth. These findings augment the study by Plowright & Pallett [11] who found that DMP colonies in *Bombus atratus *had a considerably slower rate of growth than all-worker-producing inbred colonies in laboratory conditions.

Overall colony fitness was gauged by the total number of offspring produced by the end of the experiment, as the number of reproductives reared by a colony is highly correlated with the number of workers [29,31]. The mean number of offspring produced by the diploid male colonies was significantly lower than in the other treatments. In fact, the mean was only 15.8, which is barely greater than the colony size of 15 when nests were placed in the field. The low number of offspring in these colonies would result in fewer foraging workers and hence a lower food intake. This would have initially impeded growth and subsequently led to colony starvation. This is reflected in lower survival of DMP colonies; the queens survived approximately a third of the time of the outbred colonies, and died presumably due to starvation due to the lack of foraging workers. A similar outcome has been found in the fire ant *Solenopsis invicta*, where DMP colonies had lower brood weight, fewer adult workers and higher queen mortality compared to all-worker-producing colonies [12]. This was explained by the queen having to cope on her own for longer before there were sufficient workers to take over foraging duties.

As well as reducing colony survival, bumblebee diploid males impose a genetic load on populations as they yield no reproductive return for the resources invested in them. *Bombus terrestris *diploid males produce diploid (rather than haploid) sperm. They also have smaller testes and fewer spermatozoa than haploid males, and hence have reduced fertility [32]. Although they develop normally in other respects and are capable of mating, Duchateau & Marien [32] found that the queens mated to diploid males did not produce colonies. It has since been found that such queens are capable of producing a viable colony containing triploid offspring, but the triploid queens produced are inviable or infertile [33]. Therefore, as in other species such as the sawfly *Athalia rosae ruficornis*, the costs of diploid males are not all immediately apparent, but become so a generation later [10].

Diploid males have been found to be sensitive indicators of the loss of genetic diversity in Hymenoptera. For example, an apparently abundant species of orchid bee *Euglossa imperiali *was found to have large numbers of diploid males, ranging from 12% to 100% of the total population. This turned out to be the result of an extremely low effective population size [34]. In a further study of more orchid bee species, the highest diploid male frequency and the lowest genetic variability was found in the rarest species [35]. Diploid males have also been found in rare and localised bumblebee species. In the Japanese bumblebee *Bombus florilegus*, diploid males were found in 28% of colonies produced in the laboratory from wild caught mated queens, a figure thought to be due to matched matings resulting from notably low genetic diversity and small population size. Additionally, the frequency of triploid females was found to be 2.7% in natural populations [22]. Diploid males were detected at a frequency of 5% in the wild (with respect to haploid males) in the threatened bumblebee *Bombus muscorum*, again probably due to reduced genetic diversity brought about by population fragmentation and isolation [20]. As diploid males are produced from the first brood, they will be found on the wing, even if the colony from which they have been produced dies prematurely, as our results suggest is highly likely. Because of the significant costs diploid males represent for bumblebee fitness, their frequency could potentially be used as an indicator of the genetic health of the population and hence its sustainability and conservation requirements [34]. Where the production of diploid males is high, translocations from other populations might be considered as a means of increasing genetic diversity. However, given that DMP colonies are short-lived under field conditions, their apparent absence will not always indicate a genetically healthy population. A method of directly assessing CSD allele diversity would therefore be of great value.

In this experiment the only apparent cost of inbreeding was the production of diploid males, as the non-DMP inbred colonies did not differ significantly from the outbred colonies in all the variables measured. It should be noted, however, that this lack of difference could be due to the inbred colonies resulting from only one generation of brother-sister mating, which would not substantially decrease their level of heterozygosity relative to the outbred colonies. Indeed, one study has demonstrated decreased fecundity and colony size when *B. terrestris *queens are sibmated for several generations (Beekman *et al.*, 1999). Despite the fact that some evidence indicates that Hymenoptera, including bumblebees [29,30], suffer from inbreeding depression, a meta-analysis has shown that the magnitude of fitness loss on inbreeding is less than that experienced by diploid insects [4]. This supports ideas that deleterious recessive alleles are expressed and thus purged in haploid males [3]. Our data show that the high costs of DMP following inbreeding far outweigh any apparently small effects of conventional inbreeding depression. Thus, whilst Hymenoptera may be spared some costs of inbreeding by virtue of their haplodiploidy, their sex determination system imposes unique costs through diploid male production. Due to these negative fitness effects, selection should act strongly on haplodiploids to avoid incestuous matings and the production of diploid males, a theory that has been supported by a recent study [36]. There is some evidence to suggest that this avoidance behaviour occurs through a kin recognition system [37].

Hibernation survival and colony growth in the laboratory were significantly influenced by maternal family line. This among-family variation has been found in a number of different fitness traits in bumblebees [28,29] and is evidently an important aspect of their evolutionary ecology. The factors that maintain this variation in wild populations remain to be established. Mating date was another significant predictor of the variation in hibernation survival observed; queens that were mated first were more likely to survive than those mated at a later date, despite standardised hibernation duration and conditions. This substantiates the idea that individuals that are born and reproduce early in the season have a higher survival rate and fitness [29,38].

## Conclusion

We conclude that the diploid males produced following inbreeding impose large costs on bumblebees through their influence on a colony's survival and productivity. We suggest that they act as indicators of the genetic health of the population, and therefore their detection could be an indication of genetic problems in bumblebees and other social hymenopterans. Haplodiploidy may render the social Hymenoptera less susceptible to inbreeding depression compared to diploid species, due to purging. However, our data demonstrate that the magnitude of fitness costs from DMP following inbreeding may well be as extreme as those expected to result from conventional forms of inbreeding depression in diploid species.

## Methods

### Experimental protocol

10 laboratory colonies of *Bombus terrestris*, purchased from Koppert Biological Systems, The Netherlands, in February 2008, provided young queens and males. When these sexuals emerged they were removed from the maternal colony and housed in single sex sibling groups before being mated in large mesh sided flight cages between 1st April and 16th April 2008.

To generate the outbred treatment, maternal colonies were paired randomly and daughter queens were mated with the unrelated males from their paired colony. To generate inbred colonies, daughter queens were mated with their brothers. It was expected that approximately half the inbred matings would result in diploid male colonies. Only sibling groups were used; all males in the mating cage at any one time were brothers, and all queens were sisters. Bees were mated in groups (n = 15 to 60), always in a 1:2 ratio of queens to males. During copulation mating pairs were removed from the flight cage, placed into clear plastic boxes, then left undisturbed until copulation ended.

A total of 210 queens were successfully mated (82 non-sibmated and 128 sibmated); an average of 21 ± 3.2 (mean ± SE) queens per maternal colony. After mating, males were removed and queens were kept in the box for 48 hours under natural lighting with sugar water and fresh pollen *ad libitum *(honey bee pollen stored at -20°C). After this period queens were housed individually in match boxes and hibernated in an incubator at 6°C for 47 days.

Queens that survived hibernation were placed in individual wooden boxes (10 cm × 10 cm × 10 cm) and kept under standard rearing conditions (28°C, 60% relative humidity and red light [39]). Sugar water (50% Attracker solution in distilled water, Koppert Biological Systems, The Netherlands) was provided *ad libitum *and pollen balls (ground fresh pollen mixed with Attracker) were provided three times a week. When a queen had produced five offspring, the new colony was transferred to a larger plastic box (25 cm × 22 cm × 14 cm) with a separate feeding chamber. Following Duchateau *et al. *[5], diploid male colonies were identified as those producing workers and males in approximately equal numbers from the first brood. When fifteen eclosed offspring had been produced the nest box was insulated and placed in a waterproof outer box. The colony was then transferred to the field site where the workers could forage under natural conditions. Colonies of all treatments were placed out in the field at approximately the same time. The field site was situated on the edge of Stirling University campus, from where ornamental gardens, deciduous woodland and mixed farmland were available within 500 m radius (a conservative estimate of foraging range for this species, see [40,41].

After field placement, colonies were checked weekly; on each occasion 10% of the offspring was removed and stored at -80°C for later dissection in a separate study on parasite resistance. No offspring were removed if fewer than 10 were present. When the queen died, each colony's inner brood chamber was collected and frozen for subsequent inspection.

### Variables measured

#### Hibernation survival

Following Gerloff & Schmid-Hempel [29] queens were classified as having survived hibernation only if they survived for at least 72 hours post hibernation; those that did not were unlikely to have survived under natural conditions. A considerable proportion of queens (0.108, n = 213) fell into this category. All subsequent analyses remain qualitatively unchanged if these queens are included.

#### Colony foundation

Queens were considered to have founded a colony if they successfully reared at least one offspring to adulthood. The number of days from the end of hibernation to the emergence of the first worker was recorded. Queens that had not laid eggs 12 weeks after the end of hibernation were removed from the experiment.

#### Colony growth

The number of colonies successfully rearing ≥5 and ≥15 offspring was recorded. These colony sizes were specifically relevant as at 5 offspring the colony was transferred to a larger box where the workers had to travel a short distance to find food and at 15 offspring the colony was transferred outside to forage independently.

#### Survival under field conditions

The number of weeks between the field placement date and the queen's death was recorded.

#### Final colony size

The final colony size was recorded when the queen died. For the colonies which were placed outside this was assessed by counting the number of empty cells in the brood clump. This is a reliable indirect measure of fitness as the number of reproductives produced by a colony is highly correlated with colony size [29,31]. For colonies that had not reached 15 offspring, the experiment was ended 120 days after the queen had emerged from hibernation and the final colony size was counted at this time.

### Statistical analyses

Data were analysed in SPSS 15.0 for Windows (2007 Chicago: SPSS Inc.). Binary logistic regression was used to investigate determinants of hibernation survival and colony foundation. Inbreeding treatment and maternal colony were entered as fixed factors and hibernation end date was included as a co-variate to control for variation in experiment start dates. Colony growth was analysed with binary logistic regression to assess whether or not colonies from different inbreeding treatments crossed the 5 worker and 15 worker size thresholds. Similarly, General Linear Models (GLMs) were used to investigate whether inbreeding status influenced the number of days to the emergence of a colony's 1^st^, 5^th ^and 15^th ^offspring, as well as the impact of inbreeding on colony field survival time and total number of offspring produced. In each case inbreeding treatment and maternal colony were entered as factors and hibernation end date as a covariate. All data sets used in GLMs were normally distributed (verified with Anderson-Darling tests). Variables not contributing significantly to models were removed in a step-wise fashion. Pairwise differences between factor means were investigated using Tukey's post hoc tests. Means are recorded ± their standard errors throughout.

## Authors' contributions

All authors collaboratively designed the study. PRW carried out the laboratory and field work, analysed and interpreted the results and drafted the manuscript. MCT & DG contributed to the interpretation of the data and helped to draft the manuscript. MJFB assisted with development of experimental protocols. All authors read and approved the final manuscript.
